# FAITH IS SIGNIFICANTLY ASSOCIATED TO SOCIODEMOGRAPHIC AND CLINICAL CHARACTERISTICS IN BRAZILIAN OLDER ADULTS

**DOI:** 10.1093/geroni/igad104.0930

**Published:** 2023-12-21

**Authors:** Yolanda de Souza, Angelo Jose Bós, Mariana Salecker

**Affiliations:** Pontifical Catholic University of Rio Grande do Sul, Porto Alegre, Rio Grande do Sul, Brazil; Pontifical Catholic University of Rio Grande do Sul, Porto Alegre, Rio Grande do Sul, Brazil; Pontifical Catholic University of Rio Grande do Sul, Porto Alegre, Rio Grande do Sul, Brazil

## Abstract

Religiosity is a strong factor related to health in older adults, which could positively affect physical and mental health. We aimed to observe the association between sociodemographic and clinical factors and the religiosity level in older adults. This is a secondary analysis of the Brazilian Older-adults Health Longitudinal Study (ELSI-Brazil) run by Fiocruz Institute in 2015 using a national sample of older adults (60+ year-old) population. The dependent variable was “How much the religious faith give meaning to your life?” and the independent were: sociodemographic (age-group, sex, race, marital status, and scholarly) and clinical (auto perception of health and non-transmittable chronic diseases diagnose - NTCD) characteristics, tested by Chi-Square with p<0.05 significant level. The total sample accounted for 4,669 participants, 4,080 (87.4%) answered that faith gives a lot of meaning to their lives. This percentage was significantly higher in women (91.2% vs 81.8% in men, p=0.003), indigenous race (95%, p=0.003), lower scholarly (p<0.001) and better auto perception of health (p=0.021). NTCD significantly related to higher importance of religiosity were hypertension (88.3%), dyslipidemia (90.0%), angina (93.9%), arthritis (90.5%), osteoporosis (91.0%), depression (88.7%), and cancer (90.4%). Participants with Diabetes (86.1%), pneumopathies (85.9%), and Parkinson (86.7%) had significant lower levels of religiosity. Religiosity had a significant association with sex, race, scholarly, health auto perception and NTCD, including cancer. The results evidence the important role of religious faith in the Brazilian older adult’s health. We concluded that religion and spiritual approach should always be considered to older adults.

